# Citizen science and marine conservation: a global review

**DOI:** 10.1098/rstb.2019.0461

**Published:** 2020-11-02

**Authors:** Rachel Kelly, Aysha Fleming, Gretta T. Pecl, Julia von Gönner, Aletta Bonn

**Affiliations:** 1 Centre for Marine Socioecology, University of Tasmania, Hobart, Tasmania 7005, Australia; 2 Institute for Marine and Antarctic Studies, Hobart, Tasmania 7001, Australia; 3 CSIRO Land and Water, Castray Esplanade, Hobart, Tasmania 7001, Australia; 4 Helmholtz Centre for Environmental Research - UFZ, Department of Ecosystem Services, Permoserstr. 15, 04318 Leipzig, Germany; 5 German Centre for Integrative Biodiversity Research (iDiv) Halle-Jena-Leipzig, Deutscher Platz 5e, 04103 Leipzig, Germany; 6 Institute of Biodiversity, Friedrich Schiller University Jena, Dornburger Strasse 159, 07743 Jena, Germany

**Keywords:** citizen science, marine conservation, marine ecosystems, stakeholder engagement

## Abstract

Climate change, overfishing, marine pollution and other anthropogenic drivers threaten our global oceans. More effective efforts are urgently required to improve the capacity of marine conservation action worldwide, as highlighted by the United Nations Decade of Ocean Science for Sustainable Development 2021–2030. Marine citizen science presents a promising avenue to enhance engagement in marine conservation around the globe. Building on an expanding field of citizen science research and practice, we present a global overview of the current extent and potential of marine citizen science and its contribution to marine conservation. Employing an online global survey, we explore the geographical distribution, type and format of 74 marine citizen science projects. By assessing how the projects adhere to the Ten Principles of Citizen Science (as defined by the European Citizen Science Association), we investigate project development, identify challenges and outline future opportunities to contribute to marine science and conservation. Synthesizing the survey results and drawing on evidence from case studies of diverse projects, we assess whether and how citizen science can lead to new scientific knowledge and enhanced environmental stewardship. Overall, we explore how marine citizen science can inform current understanding of marine biodiversity and support the development and implementation of marine conservation initiatives worldwide.

This article is part of the theme issue ‘Integrative research perspectives on marine conservation’.

## 1. Introduction

Climate change [1], overfishing [2], marine pollution [3] and an increasing list of other anthropogenic drivers threaten our global oceans. Many iconic marine environments, including the Great Barrier Reef in Australia and the continent of Antarctica, are nearing or have reached their critical tipping points [4] and rising ocean temperatures and sea levels are expected to further threaten marine environments and species around the globe [5]. Improved and consolidated marine conservation efforts are urgently required and consequently, the United Nations have declared the next 10 years the Decade of Ocean Science for Sustainable Development (herein, the Ocean Decade; [6]). The Ocean Decade aims to address current knowledge gaps in ocean science and build capacities for governments and communities to enable conservation action worldwide.

Marine citizen science presents a promising avenue to achieve these aims, most specifically by improving engagement and efforts in marine conservation around the globe [7]. Where properly executed, citizen science can provide robust science and high-quality data that can be used for policy and decision-making [8,9] more cost-effectively than traditional forms of science [10,11]. It can also improve public science literacy through participant learning [12,13] and sharing of knowledge among wider social networks [14]. Participation in marine citizen science enables communities to engage with the ocean, and inform themselves (and potentially their wider social networks) about issues including marine species redistributions [15], seafood harvesting [9], marine plastic pollution [16], cetacean conservation [17] and marine environmental planning [18], among others. Moreover, community-based citizen science efforts can enable more rapid implementation of research results into policies and management [19].

Citizen science, as a partnership between members of the public and academic scientists, can address scientific questions and issues of common concern [20] that foster and support innovation in science, as well as in policy and society [21]. We acknowledge that citizen science is defined, interpreted and executed in multiple ways for different purposes [8,22–25]. In this paper, we focus on citizen science for the purpose of marine conservation. Conservation initiatives that collaborate with citizen scientists and involve local stakeholders can be more effective [26] because they can engage local and traditional knowledge more readily [19], inspire conservation-minded behaviours [27], encourage relationship-building [28] and increase the legitimacy of the science and data used [29]. Citizen science is demonstrated to contribute to science knowledge gaps by using ‘people power’ to collect data; for example, in remote regions (e.g. *the Secchi Disk Project*; [30]), in real time (e.g. *Redmap Australia*; [15], https://www.redmap.org.au/; see box 1) and in high quantities (e.g. the *Australasian Fishes Project*, which has collected over 66 000 observations in less than 3 years; https://www.inaturalist.org/projects/australasian-fishes). Data obtained through citizen science can make valuable contributions to populating broad-scale databases [31] and informing natural resource management and policy [32], including the potential to monitor change in relation to the UN Sustainable Development Goals [11]. Participation in citizen science engages people with their local environments [10,33], enhances social capital [34] and encourages stewardship and marine citizenship [12,35] by providing ‘hands-on’ experiences that generate greater awareness of marine environments [16].

Marine citizen science programmes have expanded in scope and increased in number in recent years [10,14,22,36], likely as a result of the growing potential for web- and smartphone-based citizen science [23,37]. However, to date and to our knowledge, no assessment of the current extent and potential of marine citizen science for marine conservation has been conducted on a global scale, although other research has contributed to understanding the contribution of citizen science to marine science more generally [36]. Considering the imminent Ocean Decade, now is a critical time to consolidate and appraise global marine citizen science against expectations of innovation in science and policy, in order to guide the potential use of citizen science as a tool to support marine conservation. The broader citizen science community (i.e. terrestrial and marine) has already begun to professionalize and consolidate its knowledge and capacity-building, and several citizen science associations have been established in recent years in Europe (European Citizen Science Association, ECSA), Australia (Australian Citizen Science Association, ACSA) and in the USA and globally (Citizen Science Association, CSA), as well as budding initiatives in Africa and Asia [38].

**Figure 1.**
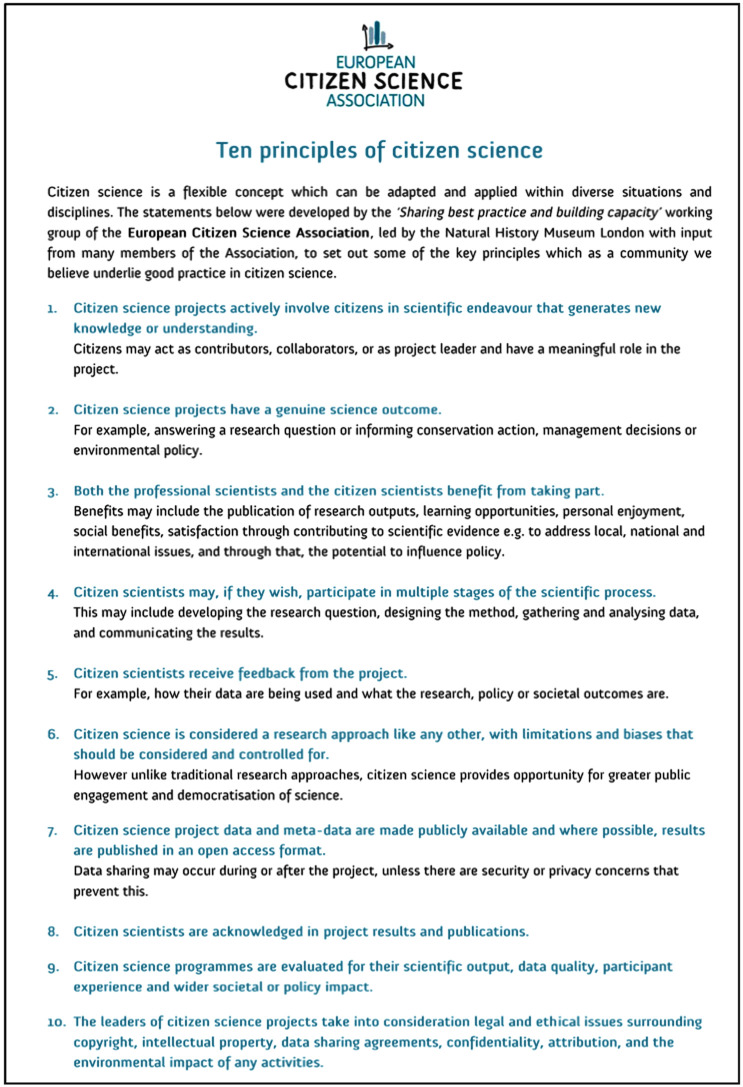
Ten principles of citizen science [39].

In order to develop citizen science as a transdisciplinary research field that can contribute at the science–society interface, as well as to act as a catalyst for the citizen science community, in 2018, the ECSA community of citizen science practitioners and researchers defined Ten Principles of Citizen Science, which seek to provide a foundation for good practice in citizen science (figure 1; [39]). The Ten Principles aim to provide a framework against which to assess excellence and underpin responsible and impactful citizen science, and they are intended to be universal, actionable and inclusive [39]. While the Ten Principles have been applied to the practical development of citizen science, these aims have not yet been evaluated, nor have the extent and impact of the principles on current citizen science projects been measured. In this study, we qualitatively assess how marine citizen science projects currently (do or do not) follow the Ten Principles, to determine whether these principles are fit for purpose in a marine context. Building on this assessment, we identify whether there is potential for the Ten Principles to further guide the development of citizen science in practice. We conduct a global survey of marine citizen science projects to (i) provide a global overview of how and where marine citizen science programmes are conducted and in which formats, (ii) evaluate these projects against the Ten Principles, and (iii) assess the potential of citizen science in enhancing current understanding of marine biodiversity and supporting marine conservation.

## 2. Methods

### (a) Survey design and implementation

In this study, we aimed to collect current data on marine citizen science projects around the world to document their extent, scientific focus and participant engagement strategies, and to investigate how the projects adhere to the Ten Principles of Citizen Science. To achieve these aims, we chose to implement an online semi-qualitative survey of marine citizen science projects that was completed by project managers, leaders and organizers. Human ethics approval for this research was granted by UFZ Datenschutz (Data Protection) in Leipzig, Germany, in July 2019. The survey questions built upon previous marine citizen science research (i.e. [35]) and were developed in the context of the Ten Principles. The survey instrument included a mix of multiple-choice (i.e. categorical), ranking (i.e. Likert scale) and open-answer questions, which sought to obtain general information about the projects (i.e. location, scientific focus, participant number), as well as their ability to develop partnerships (i.e. identify actual/realized project partnerships for funding, education, outreach). A full list of survey questions is provided in electronic supplementary material, appendix SA. The survey was piloted with citizen science project managers and scientist colleagues before it was launched on the online survey platform Survey Monkey (SurveyMonkey Inc.).

As the survey was launched as an open call to all marine citizen science projects globally, the potential for non-response bias was a factor that was considered and addressed as much as possible to mitigate any potential influence on results. First, the survey was shared and promoted online through diverse means: via direct emails to citizen science and personal networks; via citizen science newsletters; and on social media, including Twitter and Facebook. Social media in particular has been demonstrated as an effective and time-efficient recruitment strategy for online surveys [40]. Second, the survey was initially launched online for a duration of two weeks, and later extended for an additional four weeks (a total of six weeks, during July and August 2019) to allow more projects to participate, and reminders of these deadlines/extensions were communicated via email networks, newsletters and social media posts. Third, the survey instrument itself communicated the aims of the survey, identified the researchers conducting the study and included information about participant confidentiality and anonymity, in order to communicate the legitimacy and value of the survey research to citizen science projects.

### (b) Survey analysis

To determine whether there were any differences in project responses to the categorical questions across the survey sample (e.g. related to structure, funding, focus, etc.), the projects were analysed according to their participant numbers: i.e. Group 1 (*G*1; *n* = 35) = 0–99 participants; Group 2 (*G*2; *n* = 24) = 100–999 participants; and Group 3 (*G*3; *n* = 15) ≥ 1000 participants. For the purpose of this analysis, the Likert data (i.e. ranking; ranging 1–5) were analysed by calculating the ordinal means, interval modes and percentages for each of the Likert responses across all surveyed projects. As is common with online surveys, the response rates to the survey questions varied, even though care was taken to design the survey to be as brief as possible. As such, the actual response rates (*n*) of the total number of projects and percentages of agreement are accounted for in the analysis of the nominally scaled data. To test for significant differences between the three size groups (*G*1–*G*3) in the nominally scaled data, the responses to several questions were compared across the three size groups using *χ*^2^ tests. Furthermore, owing to the impossibility of avoiding non-response bias and quantifying potentially absent projects, particularly in the case of this unknown sample, the impact of missing data was not certain or quantifiable [41]. The semi-qualitative approach used here provided richer insights to describe the contextual project experiences, i.e. the quotations used below are not necessarily representative of the entire survey sample, but rather provide descriptive insights of projects' experiences and perspectives (as is typical in qualitative research) [42].

The survey data and results were used to assess the projects against the Ten Principles. Several questions specifically inquired about project adherence to a Principle (e.g. Principle #4—*Citizen scientists may participate in various stages of the scientific process*), while other results were accumulated to inform other Principles (e.g. Principle #9—*Citizen science programmes offer a range of benefits and outcomes which should be acknowledged and considered in project evaluation*). The survey participants were not specifically asked whether they believed their projects adhered to the Ten Principles, as it would be anticipated that there would be bias in response to such a question. Thus, the results described below are informed by the overall project responses.

## 3. Results

**Figure 2.**
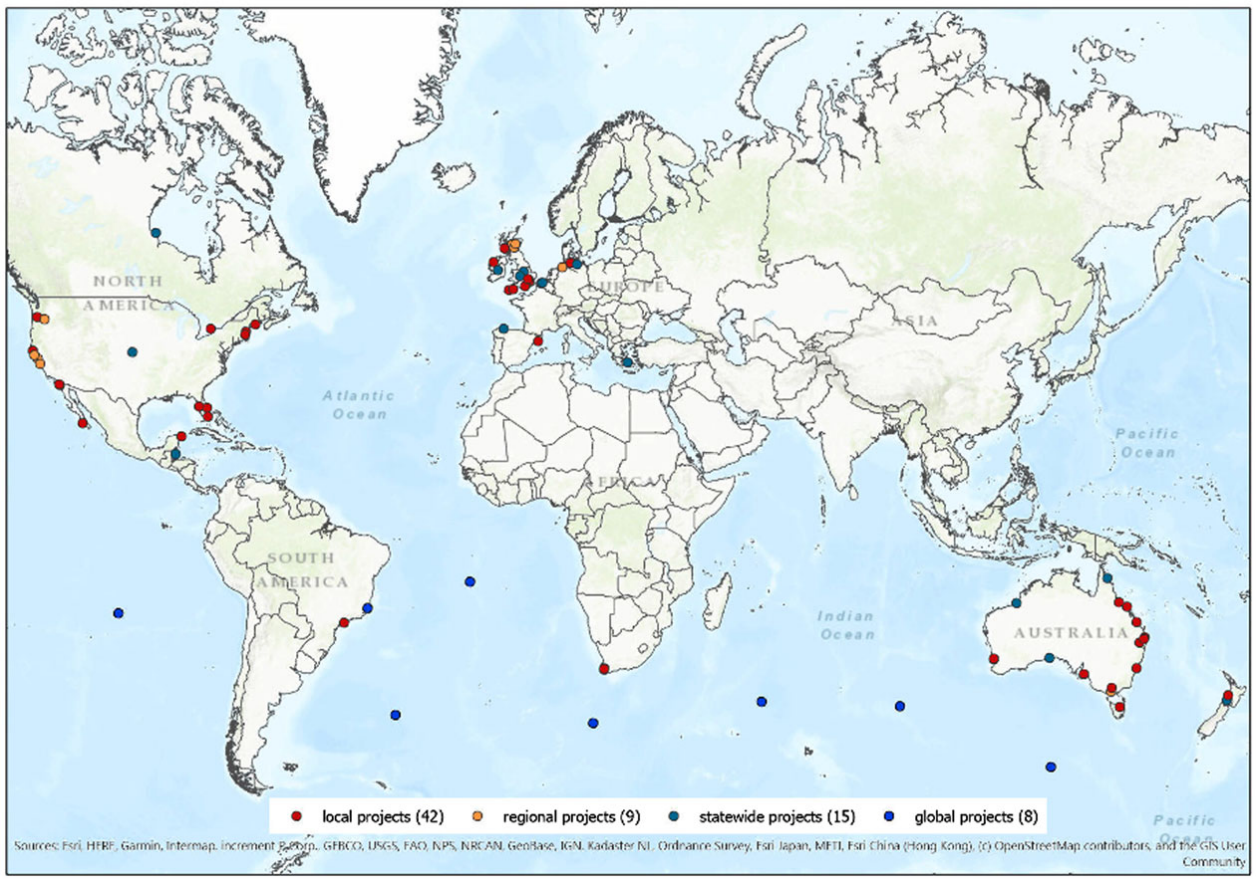
Global map of marine citizen science projects (*n* = 74; *G*1 *=* 35, *G*2 *=* 24, *G*3 *=* 15).

This study documented and analysed survey responses from marine citizen science projects to outline how, and to what extent, marine citizen science programmes follow the Ten Principles of Citizen Science, and currently contribute to marine conservation. As the survey was one of the first (to our knowledge) to conduct such an assessment of marine projects, it was impossible to construct an accurate estimate of the total number of marine citizen science projects globally; however, this study provides a baseline on which to develop further metrics. In total, responses from 74 marine citizen science projects were recorded via the online survey (excluding eight incomplete survey responses). The geographical distribution of projects was primarily clustered in Europe, North America and Australia, with some other projects located in South Africa, Latin America and New Zealand (figure 2). While some projects recorded very large numbers of up to 16 000 registered participants, the total number of active participants within each of the projects ranged from approximately 5 to 7000 participants (mean = 720; median = 200). Thus, the projects are assessed under three group sizes (*G*1, *G*2, *G*3). In the qualitative responses, several projects expressed their difficulty in recording participant numbers owing to the types (i.e. online) and frequency (i.e. one-off events such as beach-cleans, annual meet-ups, etc.) of project participation.

There was a significant difference between the three size groups in regard to the methods and approaches they used to engage with citizen scientists (*n* = 66; *χ*^2^ < 0.001***). Most of the large-scale projects (*G*2 = 83%, *G*3 = 93%) reported having a project website, while only 63% of smaller projects (*G*1) had established a project website. Similarly, smaller-sized projects were less likely to have developed a project app (*G*1 = 20%, *G*2 = 17%), while an app was common for larger-sized projects (*G*3 = 67%). Results revealed that in-person engagement was also greater in larger-sized projects; e.g. annual project meet-up events occurred more across *G*3 (40%) than *G*2 (21%) or *G*1 (17%). Significant differences between the three size groups were also found in regard to the types of training and education they provided for citizen scientists (*n* = 69; *χ*^2^ = 0.0022**). Large- and medium-sized projects more frequently used online websites as a platform for participant education (*G*2 = 71%, *G*3 = 80%) than smaller- sized projects (49%). In-person seminars and events were employed to engage project participants across all size groups (*G*1 = 54%, *G*2 = 67%, *G*3 = 47%).

The projects focused their activities mainly on coastal habitats (including rocky seashores and beaches, and open ocean areas). This result is in agreement with other research that has documented marine citizen science projects occurring in primarily easily accessible coastal habitats [36]. It was especially more common for larger projects (G3) to conduct their activities on these habitats (beaches 53%; rocky seashores 60%; coasts 73%) than smaller-sized projects (*n* = 69; *χ*^2^ = 0.0256*). Multiple other environments were identified in the qualitative responses, including coral reefs, mangroves, seagrass beds, mudflats, kelp forests, estuaries, rocky reefs, polar and aquaculture farms. This diversity of project focus has been similarly documented in other studies on terrestrial citizen science [24]. Overall, projects across all size groups targeted their data collection on specific locations or environments (66.7%; *n* = 69) and focused on species recording with identifying (65.2%) and/or quantifying marine species (56.5%). In total, 59 projects (85.5%) engaged in biodiversity monitoring activities (e.g. [38]). There was no significant difference across the three size groups in the types of data they collected (*χ*^2^ = 0.3129). The qualitative responses revealed that projects undertook diverse types of data collection, including ‘litter reporting’ (P47), *‘*measuring noise pollution’ (P50), ‘habitat-mapping’ (P23) and ‘bio-acoustic surveys' (P76).

### (a) Project partnerships and funding

In citizen science, project success and impact depend heavily on collaboration [8]. Overall, a large majority of marine citizen science projects had developed partnerships with multiple organizations (80.3%; *n* = 71) and across sectors, predominantly with civic organizations (e.g. NGOs; 62%), science (universities and marine research institutes combined; 76.1%) and government (including environmental agencies; 69%). No significant differences were identified between the three size groups (*χ*^2^ = 0.0637), although larger-scale projects (*G*3) collaborated with partners including NGOs (80%) and government (60%) more often than the smaller-scale projects (*G*1).

The projects’ capacity to engage with participants was largely determined by their funding, which came from various sources: 86.3% of projects (*n* = 73) received funding from more than one source, which included government (54.8%), universities and research institutes (combined 30.1%), environmental agencies (16.4%), NGOs (20.5%) and private enterprises (31.5%). Several projects specifically highlighted charities and philanthropic organizations as key partners and funders of their projects (15.1%), although the projects that received this type of funding were more likely to be larger in size (*G*1 = 11%, *G*2 = 8%, *G*3 = 33%). Significant differences in project funding were identified between the three size groups (*χ*^2^ < 0.001***). Larger projects (*G*3) were more likely to be funded by government and environment agencies (*G*2 = 58%, *G*3 = 60%) than smaller projects (*G*1 = 49%). In addition, nine projects (12.3%) did not receive any funding at all, and/or were self-funded.

### (b) Ten principles of citizen science

Most projects followed the Ten Principles in practice, although adherence to specific principles varied among the project sample. The survey data evidenced that the Ten Principles are largely fit for purpose in a marine context, although some results on the power dynamics in citizen science (e.g. #4) and the need for improved evaluation of citizen science projects and impacts (#9) suggest that there are key areas requiring address in marine projects overall, which we explore in more detail below.

#### (i) Principle #1: citizen science projects actively involve citizens in scientific endeavour that generates new knowledge or understanding

**Figure 3.**
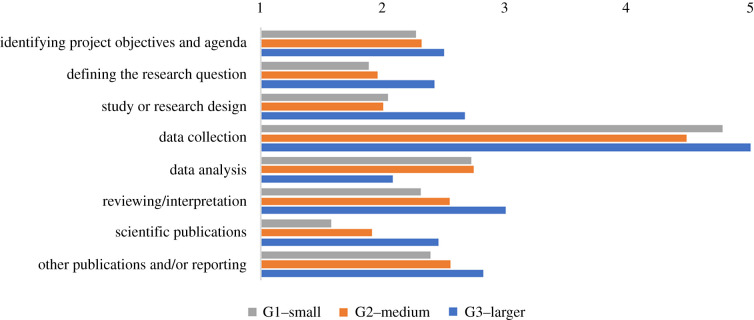
How were/are the citizen scientists involved in the scientific process? (1, not involved; 5, completely involved)*.* These responses to the Likert data questions were averaged to give a continuous (i.e. interval) score for criteria (*n* = 62; *G*1 *=* 27, *G*2 *=* 22, *G*3 *=* 13). (Online version in colour.)

The majority of respondents stated that participant involvement largely focused on data collection and citizen scientists were less involved in other project phases (figure 3). While several projects stated that citizen scientists were involved in identifying the broader project objectives, overall, the citizen scientists contributed little to project design or project output phases. Thus, it can be inferred that most projects are contributory, and only a few of the projects surveyed were co-created projects (e.g. *Friends of Gulf of St Vincent*, Australia; *Community Marine Biodiversity Monitoring*, Scotland). It is noteworthy that several projects (*n* = 7) stated that their citizen science participants were not involved in the scientific process, even though their participants were reported to be involved in data collection activities.

#### (ii) Principle #2: citizen science projects have a genuine science outcome

Most but not all projects (73%; *n* = 58) confirmed that they are working to achieve a genuine scientific outcome, although this was more likely in medium-sized projects (*G*1 = 69%, *G*2 = 96%, *G*3 = 73%; *χ*^2^ = 0.0167*). Significant differences were found between the types of scientific outcomes recorded by the three size groups (*χ*^2^ = 0.00218**). Smaller-sized projects (*G*1) were less likely to produce data than the larger projects (*G*1 = 69%, *G*2 = 92%, *G*3 = 87%), and medium-sized projects (*G*2) were more likely than the other groups to publish scientific articles (*G*1 = 40%, *G*2 = 80%, *G*3 = 47%). However, the qualitative responses revealed that project outcomes varied overall across the size groups, with extensive direct and indirect contribution to peer-reviewed literature, e.g. *Open Litter Map* [43], the *Secchi Disk Study* [30], *Reef Life Survey* [44] (https://reeflifesurvey.com/; see box 1), *Wildbook for Whale Sharks* [45], *Send Us Your Skeletons* [46], the *Ocean Microbiome Project* [47]; as well as contributions to data records and repositories, and development and testing of new methodologies, e.g.:

Generation of large photographic catalogues of manta ray individuals and sighting reports, increasing our ability to examine their ecology (P9).

We are collecting baseline data to measure the erosion and accretion of sand on beaches, and assessing seasonal variation versus net loss/gain. The main outcome is providing data to the government to develop long-term strategies for the coastline (P16).

#### (iii) Principle #3: citizen science provides benefits to both science and society

The project respondents revealed that citizen science provided a range of benefits to science across the project group sizes. In regard to scientific advances, data collection was perceived as most important, followed closely by scientists' engagement with the public (electronic supplementary material, appendix SB). The larger-sized projects benefited more from the production of scientific publications arising from results of large-scale data collected in space and time through citizen science (*G*1 = 23%, *G*2 = 30%, *G*3 = 33%). When asked whether their projects provided any benefit for citizen scientists, project respondents across all size groups indicated that the citizen science participants mostly benefited from knowledge exchange and learning, partaking in a fun activity, and becoming part of a community, e.g.

Development of science skills. Provides a tool for community groups to engage in environmental action (P60);

Being a part of a community with shared interests, encouraging citizen scientists to engage with nature and to observe/collect data on trends in their locations (P76).

Publication output ranked lowest and was perceived as a medium to low benefit for citizen scientists. Engagement with science scored highly, an interesting result when considering that participants were generally not actively involved throughout the whole scientific process (i.e. figure 3). Furthermore, several projects identified the trade-offs between achieving benefits for science as well as their participants, e.g.

This was the biggest challenge from my point of view. We identified many research questions we wanted to answer, but we had to remove many because the data collection was ‘too boring’ or ‘not engaging enough’. We also had to choose projects that were ‘not time bound’ to accommodate [participants'] holidays, weekdays, and include a good range of fun and diverse data collection activities (P6);

We're trying to establish a balance. But as a consequence of this, and because we are studying ‘non-charismatic’ organisms, our trainings are longer and more complex than other ones (P22);

Ensuring participant satisfaction is our highest priority, and thus if scientific goals are not met, we send a team of scientists after the citizen science field work to complete tasks that were not finished (P29).

#### (iv) Principle #4: citizen scientists may participate in various stages of the scientific process

As revealed above, the projects engaged citizen scientists during various stages of the scientific process, but most participation centred around data collection (figure 3). When comparing across project size groupings, data collection was still the primary form of scientific contribution from participants (*G*1 = 92%, *G*2 = 70%, *G*3 = 100%). Analysis of the Likert data revealed that citizen scientists' participation and contribution in other stages of the scientific process was poor. However, several project coordinators highlighted their intention to involve citizen scientists more actively through the entire scientific process to develop project design and better engender ‘two-way exchange between citizens and researchers’ (P1). Other projects highlighted that their participants communicated project outcomes to the wider community (i.e. science dissemination), which corroborates other research that has demonstrated the role citizen scientists play in communicating the science they learn to wider community groups [14], e.g.

Science communication and sensibilization to society (P2);

Participants are very involved in development of exhibits about the project, reporting back to the community (P60).

#### (v) Principle #5: citizen scientists receive feedback from the project

Almost all projects indicated that they provided project results (98.3%; *n* = 58) to the citizen scientist participants. Here, medium- and larger-sized projects (*G*2 and *G*3) were more likely to provide this feedback to their participants than smaller projects (*G*1; *χ*^2^ = 0.0178*; electronic supplementary material, appendix SB). This feedback was shared by projects mostly via newsletters, websites and social media. More personal feedback avenues, such as personal interaction or project meetings, were reported less often across all project size groups. The qualitative responses revealed other ways in which projects shared their results with participants; these included project reports, presentations and other project events, as well as communicating more widely via press releases or local media.

#### (vi) Principle #6: citizen science, as with all forms of scientific inquiry, has limitations and biases that should be considered and controlled for

Other research has documented that about half of marine citizen science projects include quality control in their data collection/analysis activities [36]. The results of this survey revealed that the project sample overall largely did consider and control for limitations and biases in their scientific activities (78%; *n* = 58). Smaller projects (*G*1) indicated that they were more likely to control for scientific biases during the scientific activity (i.e. with citizen science participants) than the other-sized groups (electronic supplementary material, appendix SB), likely as a result of increased opportunity for contact in more intimate project sizes. Overall, most projects did conduct checklists and standardized protocols as part of their scientific tasks. However, participant surveys were the least conducted control used across all groups, particularly larger-sized projects (score), which would infer that participant bias was not accounted for and these projects are likely semi-structured in design [48].

#### (vii) Principle #7: project data/metadata are publicly available and results are published open access

In citizen science, as with any scientific approach, rigorous data management must be implemented to ensure data quality, transparency and access [8]. Overall, most projects ensured that their data were accessible to citizen scientists (90.6%; *n* = 53), the public (83%) and other scientists (86.8%). Comparison across the project group sizes revealed that medium- and large-sized projects were more likely to publish their results in open-access formats (*G*1 = 49%, *G*2 = 63%, *G*3 = 67%; *χ*^2^ = 0.00146**). The qualitative responses revealed that many projects made their data open access on their websites (e.g. *Beach Explorer*) or in reports (e.g. *ORCA Marine Mammal Surveyors*). Other projects shared their data with governments that used it to inform marine management (e.g. *Send Us Your Skeletons*). Some projects had the capacity to also share their data in open repositories (e.g. the Atlas of Living Australia, Global Biodiversity Information Facility, etc.), which provide important technical infrastructure for citizen science [49]. A majority of the total project sample published the results of their projects' work in open-access formats (75%; *n* = 56) and many published in open-access scientific journals. The qualitative responses revealed that the majority of these made their results available on their project websites, e.g.:

Data is public and available on the web (P2);

The data is open and available to all (P47);

Publicly available on the website (P52).

#### (viii) Principle #8: citizen scientists are suitably acknowledged by projects

Most projects acknowledged their citizen science participants in their project outcomes (86%; *n* = 57), via scientific publications, annual project and government reports, in media outputs, and on the project websites. Projects across all size groups were likely to acknowledge their participants on multiple platforms (*G*1 = 87.5%, *G*2 = 76.2%, *G*3 = 100%). However, the individual citizen scientists were predominantly acknowledged collectively and anonymously because of issues around identity data protection and the difficulty in acknowledging the large numbers of participants contributing. Furthermore, the project coordinators expressed difficulty in acknowledging specific contributions because many citizen scientists are anonymous within the projects, either because of ethical reasons or owing to the contributory nature of many projects; *‘*There are too many volunteers to list so they are acknowledged openly but also anonymously’ (P18).

#### (ix) Principle #9: citizen science programmes offer a range of benefits and outcomes which should be acknowledged and considered in project evaluation

The evaluation of citizen science outcomes is typically a high priority for projects, but is also often rated as a key challenge [50,51]. The survey results largely reflected this experience. Overall, projects aimed to evaluate their outcomes, mainly for data quality. However, participant experience and wide social impact were rarely assessed and formal evaluation of projects was not typically undertaken. Smaller-sized projects (*G*1 = 51%) were less likely to conduct project evaluation than larger projects (*G*2 = 75%; *G*3 = 60%; *χ*^2^ = 0.01468*). Owing to the already considerable length of the online survey, a more in-depth assessment of the types and depth of project evaluation was not possible. Furthermore, only a few projects provided additional qualitative comments. For example, some projects highlighted that additional data photos were used to check species identification, while other projects revealed that more project evaluation was ‘a work in progress’ (P32) that was desirable but not feasible to date owing to time constraints and other limitations (for full list, see electronic supplementary material, appendix SB).

#### (x) Principle #10: leaders of projects address legal and ethical considerations of the project

A very high percentage of the overall sample of citizen science projects indicated that they considered legal and ethical issues in their project design (89.3%; *n* = 56). Overall, the primary issues considered were data sharing (71.2%; *n* = 52), confidentiality (67.3%) and the environmental impacts of the project activities (63.5%). However, when looking across the project size groups, results revealed that larger-sized projects considered legal and ethical issues more frequently than smaller projects (*G*1 = 57%; *G*2 = 79%; *G*3 = 80%; *χ*^2^ = 0.000149***). Furthermore, these larger-sized projects particularly considered data sharing and confidentiality issues (*χ*^2^ = 0.0377*; electronic supplementary material, appendix SB). The qualitative responses also highlighted ‘animal ethics’ (P14), ‘participant safety’ (P18) and ‘indigenous consultation’ (P73) as issues that projects needed to consider within their project remit.

### (c) Contribution to conservation

The majority of projects indicated that they believed their citizen science activities contribute to informing marine management (94.3%; *n* = 53), with no differences detected between the three size groupings (*χ*^2^ = 0.3864). However, projects from all size groups felt that their project results and outcomes were not used to maximum potential for science, society and/or policy (85.5%; *n* = 55). The projects' contribution to management was primarily through providing data, although some projects identified opportunity for participant discussion and dialogue with other stakeholders (i.e. marine managers) as an influence on management (*n* = 4). The actual contribution afforded by each project varied widely across the survey sample (see box 1), as was apparent in the qualitative response results, e.g.:

Box 1. **Marine citizen science in practice. Different modes of participation can facilitate conservation science and management impact.**

*Redmap Australia* (Range Extension Database and Mapping Program, https://www.redmap.org.au/) is a national citizen science project aiming to:

(i) provide an early scientific indication of climate-induced shifts in marine species distribution, and

(ii) engage the Australian public on marine climate change issues, by using their own data [14,15].

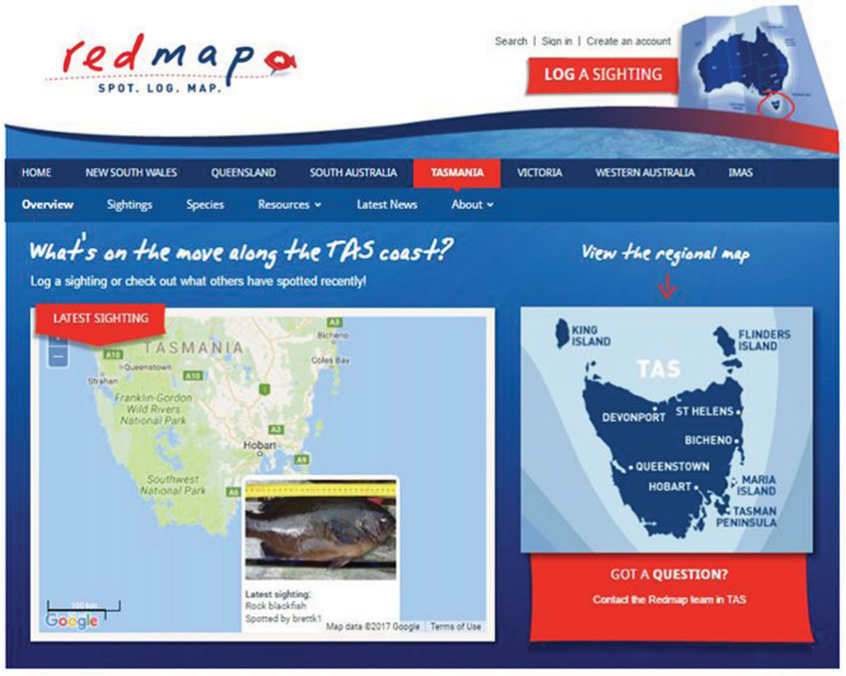

The project employs an ‘opportunistic’ citizen science approach, by inviting recreational fishers and divers to submit photographs of species they happen to observe or catch outside the species' known or expected geographical distributions. Each observation is verified by one of 80 scientific experts through the project's semi-automated verification system. Over the past decade, approximately 1000 participants have submitted photographs to the Redmap programme, resulting in at least 25 peer-reviewed scientific publications improving current understanding of marine climate change (e.g. [52–54]). In addition, several thousand people have been involved in public engagement on climate change issues, by sharing information generated by the project, e.g. via Facebook, Twitter or Web-newsletter, and by participating in community events [15].

*Snapshot Cal Coast* (https://www.calacademy.org/calcoast) in the USA is another opportunistic, contributory project that adopts a ‘bioblitz’ type approach using the iNaturalist platform (https://www.inaturalist.org/), for two weeks each year, to document ecology of the Californian coast. While it is difficult to generate exact participant numbers, this project had a significant marine conservation impact by increasing the capacity to use citizen science observations to understand and monitor biodiversity across California's Marine Protected Area (MPA) network. Specifically, this project gathers data needed to determine current species ranges, against which future changes can be measured and monitored.

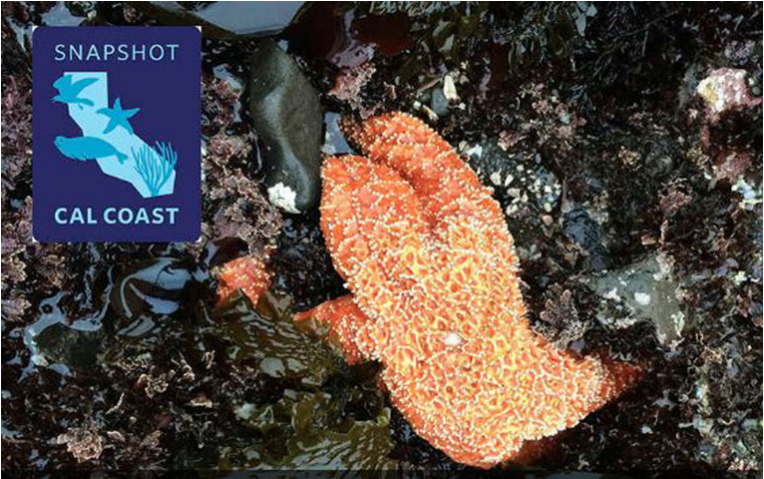

*Reef Life Survey* (https://reeflifesurvey.com/), in comparison, is a global project established in 2008 that engages highly trained citizen science divers to conduct structured, scientifically rigorous underwater-surveys of tropical and temperate reef habitats to document biodiversity [55]. Since its inception, the project has engaged with more than 260 diver participants to conduct 12 000 dives in over 50 countries, resulting in 77 scientific papers and management reports (see https://reeflifesurvey.com/scientific-papers-management-reports/).

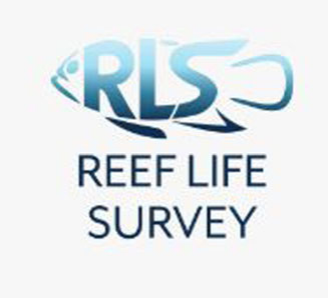

The *Studland Tagging Project* (https://www.theseahorsetrust.org/conservation/studland-tagging-project/) operates at a more local level and has a much smaller geographical scope and scale. The project runs under The Seahorse Trust, which is an umbrella NGO that aims to protect and preserve the natural environment. The Studland Tagging Project is a grassroots programme that focuses on documenting and protecting a population of seahorses in Studland Bay, UK. Citizen scientist participants include locally trained divers who engage in specific project data collection analysis (i.e. similar to Reef Life Survey), as well as opportunistic participants who submit photos of seahorses they have seen to the project (i.e. comparable to Redmap). This project has been very successful in regard to marine conservation, using their survey data to inform and secure the establishment of a marine conservation zone to protect the seahorses in Studland Bay in 2019.

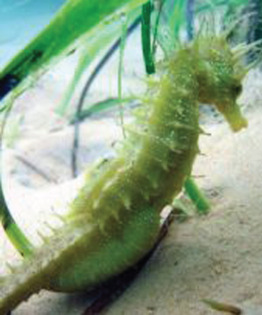

Credit: Studland Tagging Project.

(i) data

Data are being used in the 2022 MPA management review (P21);

Data collected by CitSci volunteers are used to guide management actions, including [species] removal efforts and regulations to prevent further spread (P51);

Data are accessed by scientists and government agencies and used in reporting (P52).

(ii) stakeholder discussion and dialogue

Management is very involved from scientific and policy perspectives on determining the future of the project and project goals (P29);

Collaboration with management agencies to discuss policies and procedures for active reef restoration (P30).

Other more specific (i.e. yes/no) questions explored project collaborations and contributions to marine conservation; 60% of coordinator responses (*n* = 28) indicated that their project was part of a larger conservation management initiative, although this actually represented only 23% of the survey sample overall.

## 4. Discussion

This study documented and analysed survey responses from 74 marine citizen science projects to outline how marine citizen science programmes follow the Ten Principles of Citizen Science, and currently contribute to marine conservation. The results revealed that most marine citizen science projects centre around biological and ecological data collection (in different marine environments), which corroborates observations made in other large-scale studies on citizen science in the terrestrial realm [13,23]. The heterogeneity of projects documented in this study is consistent with other research that highlights the difficulty in succinctly classifying citizen science [24], and which we argue highlights the value of citizen science in contributing to marine conservation via diverse means and formats [7].

### (a) The Ten Principles of Citizen Science in practice

While the majority of projects followed the Ten Principles in practice, adherence to specific principles varied. For example, results of adherence to principles that stipulate active citizen involvement in projects (#1), and at different stages of the research (#4), demonstrated that the projects overall were largely contributory, and focused predominantly on data collection (regardless of project size), as similarly reported in studies on terrestrial and freshwater citizen science projects [23,24]. However, while a broadening of citizen scientist participation might be a welcome option, the observed pattern of participation may also reflect the preferred mode of engagement for many participants [56].

In addition, and as noted in other studies, publication rates of project data were generally low (with some notable exceptions, e.g. *Reef Life Survey*) [57]. This would suggest poor adherence to principle #2, although the survey responses indicated that projects generally felt that they had achieved a genuine scientific outcome. The projects reported that their citizen science provided many benefits to participants (#3), but they did not consider production of scientific results as an important benefit for participants. Other research has determined that citizen scientists want their information and/or participation activities to contribute to scientific outcomes [58]; thus, the capacity or opportunity to publish data or results achieved through citizen science is a driver for citizens to participate. While publishing might not necessarily be of benefit to participants, fostering scientific advances can be a strong driver to participate in citizen science and is important to consider when identifying means to include participants more actively in the scientific process (#4).

Project size may have some influence, as larger projects generated more data and are likely to have a greater capacity to publish [59], as well as provide regular feedback to citizen scientists (#5). In addition, the survey results may be limited by the time-lags inherent in the scientific process, including delays associated with publishing. Furthermore, citizen science is an emerging scientific field that still needs to develop standardized means to combine demands on co-creation and open participation in achieving scientific success. Regardless, if the potential for marine citizen science to generate knowledge is to be fully realized, projects will need to work at enhancing capacities to more effectively share data and results with science and policy through publication and other outputs [13].

Overall, principles that focus on acknowledging citizen science contributions (#7), data sharing (#8) and consideration of legal and ethical issues (#10) were largely adhered to; again, larger projects were revealed to have greater capacities and were more likely to report that they were able to achieve these aims. Project size was also an important indicator of quality control (#6, #9). Smaller projects were more likely to provide in-depth quality control measures before, during and after data collection; e.g. through in-person training, printed and online training tools, or photo ID templates (with some notable exceptions, e.g. *Reef Life Survey*). This suggests an apparent trade-off between balancing the size of a project and its capacity to collect usable data, while also ensuring the quality of that data (#6) and participant satisfaction (#9). Joint project design with community members could provide the potential for improvement in regard to these issues. Applying statistical modelling to account for uncertainties was not yet widely used across projects; however, new tools for data integration are increasingly being developed [60,61].

### (b) Marine citizen science for marine conservation

The participatory and research-oriented structure of citizen science facilitates its alignment for supporting conservation goals and research [62]. Citizen science challenges how science is discovered and used to create new knowledge [63]. There is significant scope for citizen science to contribute to conservation approaches [59], and considerable opportunity for local people to become involved in collecting relevant data and/or conducting investigations for marine conservation [32]. In order for society to fully comprehend science and its role in conservation, people need to engage more fully with science and conservation and experience it in their daily lives [27]. Different citizen science models and approaches can contribute to different types of conservation outcomes [59]. For example, co-created projects that involve local stakeholders can facilitate buy-in, help build trust and increase collaboration between science and communities [64]. Co-created projects can also bolster local conservation action by encouraging participant connection with local issues, and providing education opportunities that enhance awareness and understanding [65].

While no model or format of citizen science is ‘better’ than another, they may vary in their capacity to contribute to conservation in different ways [59]. The majority of citizen science projects recorded in the survey were contributory in nature, and this might influence how citizen science can support marine conservation in the Ocean Decade. A major reason for this dominant approach may be that these projects are easier to create and conduct and facilitate engaging large numbers of participants at relatively low costs [13], and that potential citizen science participants are primarily interested in engaging with opportunities for data collection [66]. Larger-scale contributory projects (e.g. *Redmap*, box 1), as documented in this survey, can generate large spatial and temporal datasets that can inform academic research and indirectly contribute to conservation [27]. Furthermore, these contributory projects may also act as monitoring programmes (e.g. *MPA Watch*) that can directly inform science and conservation policy [10].

Although contributory projects dominate in this survey, some stand-out collaborative and co-created citizen science projects were recorded (e.g. the *Studland Tagging Project*, box 1). Collaborative and co-created projects may facilitate more in-depth and two-way interaction between scientists and participants, and thus support greater learning and result in enhanced civic engagement [59]. In this way, citizen science can provide a bridge between science and society [67]. Participation in marine citizen science can improve learning by ‘doing’, and enhance marine citizenship, stewardship and ocean literacy [8,35,68]. Citizen science can improve participants' self-efficacy around conserving the ocean, when their actions contribute to marine conservation and citizen scientists potentially feel empowered to contribute further to marine conservation and sustainable use [34].

### (c) Power dynamics and partnerships

The power relations and dynamics apparent in citizen science programmes are largely understudied, and there is considerable scope to improve understanding around power in citizen science (for both research and practice), particularly in a marine context. Primarily, these dynamics stem from levels of knowledge or ‘expertise’ as scientists typically establish and lead projects [63,69], further evidenced in this study. However, emerging research demonstrates that citizen scientists can develop into ‘experts’ when scientists enable them to engage and communicate as equals [70], and creating ways for citizen scientists to engage in multiple aspects of the research process may foster more collaborative and equal programme structures [71]. For example, methodology is often a point where translation between different actors in citizen science limits the quality of participation but working to co-develop methods with participants can allow more active (and a greater amount of) input and engagement [72]. Allowing participants to influence the research questions and actively contribute to science outcomes can instil feelings of ownership and responsibility for the project and its ability to achieve its aims [73], and we suggest that ownership may also arrive from other aspects such as engagement in promotion, dissemination and communication about the project and its results. Improving (ocean) science literacy can support social empowerment and community engagement in decision-making [68]. Projects wishing to develop meaningful relationships with their participants and encourage connections to the ocean should work proactively to understand and address participants' needs and objectives in engaging with marine citizen science [74].

Similar to other assessments of citizen science [13], the majority of citizen science projects collaborated with one or more partners, e.g. universities, NGOs, government, in the context of marine conservation, and these can further science engagement and awareness among broader community groups and stakeholders [67]. Recent research highlights the role of partnerships in creating meaningful collaborations that can more effectively address environmental issues [62]. Citizen science partnerships can enable wider communication and uptake of data [8], improve funding capacity, improve communication and trust between science and the public [36], and enable citizen scientists to contribute to decision-making processes in marine conservation and management that might otherwise exclude them [7]. Just over half of the projects in this survey made their data available as open access, shared it in open repositories and/or directly provided it to management. Citizen science projects can benefit greatly from developing broad partnerships, increasing participant recruitment, public engagement, data sharing and access to funding [35].

### (d) Study limitations highlight future directions for marine citizen science practice and research

This study is one of the first to identify the scale and scope of marine citizen science projects globally with a view to inform their contribution to marine conservation, and as is inherent to any horizon scanning study, it is subject to several limitations. The surveyed projects are predominantly representative of Western regions (i.e. North America, Europe and Australia), which might reflect sample bias in our survey promotion methods. The survey was shared online via citizen science and colleagues’ networks and projects not part of these networks or partnering with member groups or projects might have been excluded. However, this report is our best efforts at an accurate reflection of the spatial pattern of global citizen science and is largely supportive of the findings of other assessments (e.g. [36]). In this regard, our survey results highlight a need to create and extend a network of marine citizen science projects globally, to improve the exchange of relevant support and information and enhance project capacity, particularly in underrepresented regions, including Asia and Africa [38].

More widespread and global activity and interaction are required if citizen science is to contribute to achieving the objectives of the Ocean Decade, especially in improving capacity advancement in developing and least developed countries [6]. In particular, efforts are needed to create and extend platforms to communicate evidence-based practices of citizen science (that are currently emerging through research, evaluation and experience) more widely [67]. The budding network of marine citizen science projects documented in this study would benefit greatly from increased opportunity to communicate, share and learn from the diverse citizen science experiences revealed here. Future development of a ‘Global Marine Citizen Science Network’ will be critical to achieve such communication and knowledge-sharing goals.

Finally, the survey predominantly engaged with citizen science coordinators and managers, and future research could explore citizen science participants' views and experiences in more depth. Specifically, it would be useful to assess how participation in citizen science might bolster conservation by collaborating with communities, and enabling them to engage in and influence conservation initiatives for both land and sea [8,35,75]. The projects overall focused on ecological and biological parameters, and there is considerable potential for marine citizen science to contribute to social and socioecological-focused parameters [23] in improving understanding of and relationships with communities in regard to marine conservation. We recognize that for a global study of this nature, an online survey was the only practical method. Future studies may garner new insights by extending this work with more detailed data collection, including interviews with project managers and participants.

## 5. Conclusion

This study documented the current global distribution and scope of marine citizen science projects to inform its potential for (i) contributing to the aims of the UN Ocean Decade and (ii) connecting projects and participants for marine conservation. The results revealed that most marine citizen science projects centre around biological and ecological data collection (in different marine environments), which corroborates observations made in other large-scale studies on citizen science in the terrestrial realm [13,23]. The heterogeneity of projects documented in this study is consistent with other research that highlights the difficulty in succinctly classifying citizen science [24], and which, we argue, highlights the value of citizen science in contributing to marine conservation via diverse means and formats [7].

The survey revealed that the Ten Principles of Citizen Science are (and can be) appropriate to guide the development of citizen science in a marine context. Many projects already adhere to the Ten Principles in practice, although the survey identified key areas where the Principles need to be operationalized with guidance and evaluation. There is certainly scope for improvement in regard to engaging participants along the entire scientific process, increasing contribution to scientific publications and guidelines, and implementing thorough project evaluation and wider project communication. In this regard, frameworks are already being developed (e.g. [50,51]) to provide practical, hands-on evaluation tools that can more easily monitor and track citizen science progress and development. Such evaluation guidance could also be useful for projects in their design stages, as well as for informing funding decisions. The growing experience of citizen science projects worldwide can now serve as a solid foundation from which to enhance best practice through evaluation guidelines.

This study shows that marine citizen science comprises many different approaches and outlines great potential for marine citizen science to (continue to) enhance current understanding of marine biodiversity and support marine conservation. Marine citizen science is growing and expanding globally and is providing innovative avenues to generate new scientific data and increase public engagement in marine science and conservation. In addition to contributing data to science and management, citizen science projects need to be recognized for their role in connecting people to the oceans and furthering global ocean literacy. By generating awareness and providing opportunities for learning, marine citizen science projects can contribute to achieving the aims of the UN Decade of Ocean Science for Sustainable Development by improving engagement in tackling the intensifying challenges facing the world's oceans.

## Supplementary Material

Appendix A - Survey questionnaire. All questions of the online survey.

## Supplementary Material

Appendix B - Summary of survey results. Additional figures and tables for analysis results for the Ten Principles.

## Data accessibility

See electronic supplementary material, appendix SB for all survey results.

## References

[1] Pecl GT et al. 2017 Biodiversity redistribution under climate change: impacts on ecosystems and human well-being. Science 355, eaai9214 (10.1126/science.aai9214) 28360268

[2] Rousseau Y, Watson RA, Blanchard J, Fulton EA 2019 Evolution of global marine fishing fleets and the response of fished resources. Proc. Natl Acad. Sci. USA 116, 12 238–12 243. (10.1073/pnas.1820344116) PMC6589657 31138680

[3] Vince J, Stoett P 2018 From problem to crisis to interdisciplinary solutions: plastic marine debris. Marine Policy 96, 200–203. (10.1016/j.marpol.2018.05.006)

[4] Lenton TM, Rockstrom J, Gaffney O, Rahmstorf S, Richardson K, Steffen W, Schellnhuber HJ 2019 Climate tipping points—too risky to bet against. Nature 575, 592–595. (10.1038/d41586-019-03595-0) 31776487

[5] IPCC. 2019 Special report on the ocean and cryosphere in a changing climate. In IPCC Working Group II/IPCC Secretariat See https://www.ipcc.ch/site/assets/uploads/sites/3/2019/2011/SROCC_FinalDraft_FullReport.pdf.

[6] UN. 2019 See https://www.oceandecade.org/.

[7] Cigliano JA, Meyer R, Ballard HL, Freitag A, Phillips TB, Wasser A 2015 Making marine and coastal citizen science matter. Ocean Coast. Manage. 115, 77–87. (10.1016/j.ocecoaman.2015.06.012)

[8] McKinley DC et al. 2017 Citizen science can improve conservation science, natural resource management and environmental protection. Biol. Conserv. 208, 15–28. (10.1016/j.biocon.2016.05.015)

[9] Warner KA, Lowell B, Timme W, Shaftel E, Hanner RH 2019 Seafood sleuthing: how citizen science contributed to the largest market study of seafood mislabeling in the US and informed policy. Marine Policy 99, 304–311. (10.1016/j.marpol.2018.10.035)

[10] Hyder K, Townhill B, Anderson LG, Delany J, Pinnegar JK 2015 Can citizen science contribute to the evidence-base that underpins marine policy? Marine Policy 59, 112–120. (10.1016/j.marpol.2015.04.022)

[11] Fritz S et al. 2019 Citizen science and the United Nations sustainable development goals. Nat. Sustain. 2, 922–930. (10.1038/s41893-019-0390-3)

[12] Crall AW, Jordan R, Holfelder K, Newman G, Graham J, Waller DM 2012 The impacts of an invasive species citizen science training program on participant attitudes, behaviour and science literacy. Public Underst. Sci. 22, 745–764. (10.1177/0963662511434894) 23825234

[13] Turrini T, Dörler D, Richter A, Heigl F, Bonn A 2018 The threefold potential of environmental citizen science—generating knowledge, creating learning opportunities and enabling civic participation. Biol. Conserv. 225, 178–186. (10.1016/j.biocon.2018.03.024)

[14] Nursey-Bray M, Palmer R, Pecl GT 2018 Spot, log, map: assessing a marine virtual citizen science program against Reed's best practice for stakeholder participation in environmental management. Ocean Coast. Manage. 15, 1–9. (10.1016/j.ocecoaman.2017.10.031)

[15] Pecl GT et al. 2019 Redmap Australia: challenges and successes with a large-scale citizen science-based approach to ecological monitoring and community engagement on climate change. Front. Mar. Sci. 6, 349 (10.3389/fmars.2019.00349)

[16] Zettler ER, Takada H, Monteleone B, Mallos N, Eriksen M, Amaral-Zettler LA 2017 Incorporating citizen science to study plastics in the environment. Anal. Methods 9, 1392–1403. (10.1039/C6AY02716D)

[17] Matear L, Robbins JR, Hale M, Potts J 2019 Cetacean biodiversity in the Bay of Biscay: suggestions for environmental protection derived from citizen science data. Mar. Policy 109, 103672 (10.1016/j.marpol.2019.103672)

[18] Jarvis RM, Bollard Breen B, Krägeloh CU, Billington DR 2015 Citizen science and the power of public participation in marine spatial planning. Mar. Policy 57, 21–26 (10.1016/j.marpol.2015.03.011)

[19] Danielsen F, Burgess ND, Jensen PM, Pirhofer-Walzl K 2010 Environmental monitoring: the scale and speed of implementation varies according to the degree of people's involvement. J. Appl. Ecol. 47, 1166–1168. (10.1111/j.1365-2664.2010.01874.x)

[20] Shirk J, Bonney R 2015 *Citizen Science Framework Review: Informing a Framework for Citizen Science within the US Fish and Wildlife Service*. Ithaca, NY: Cornell Lab of Ornithology. (https://ecos.fws.gov/ServCat/DownloadFile/56072?Reference=52383).

[21] Hecker S, Haklay M, Bowser A, Makuch Z, Vogel J, Bonn A 2018 Citizen science—innovation in open science, society and policy. London, UK: UCL Press.

[22] Roy HE, Pocock MJO, Preston CD, Roy DB, Savage J, Tweedle JC, Robinson LD 2012 Understanding citizen science & environmental monitoring. NERC Centre for Ecology & Hydrology and Natural History Museum. Final report on behalf of UK-EOF. Wallingford, UK: Centre for Ecology & Hydrology.

[23] Kullenberg C, Kasperowski D 2016 What is citizen science?—A scientometric meta-analysis. PLoS ONE 11, e0147152 (10.1371/journal.pone.0147152) 26766577 PMC4713078

[24] Pocock MJO, Tweedle JC, Savage J, Robinson LD, Roy HE 2017 The diversity and evolution of ecological and environmental citizen science. PLoS ONE 12, e0172579 (10.1371/journal.pone.0172579) 28369087 PMC5378328

[25] Hecker S, Wicke N, Haklay M, Bonn A 2019 How does policy conceptualise citizen science? A qualitative content analysis of international policy documents. Citizen Sci. Theory Pract. 4, 32 (10.5334/cstp.230)

[26] Fulton S, Caamal-Madrigal J, Aguilar-Perera A, Bourillón L, Heyman WD 2018 Marine conservation outcomes are more likely when fishers participate as citizen scientists: case studies from the Mexican Mesoamerican Reef. Citizen Sci. Theory Pract. 3, 1–12. (10.5334/cstp.118)

[27] Ballard HL, Robinson LD, Young AN, Pauly GB, Higgins LM, Johnson RF, Tweddle JC 2017 Contributions to conservation outcomes by natural history museum-led citizen science: examining evidence and next steps. Biol. Conserv. 208, 87–97 (10.1016/j.biocon.2016.08.040)

[28] Kelly R, Fleming A, Pecl GT 2019 Citizen science and social licence: improving perceptions and connecting marine user groups. Ocean Coastal Manage. 178, 104855 (10.1016/j.ocecoaman.2019.104855)

[29] Ottinger G 2010 Buckets of resistance: standards and the effectiveness of citizen science. Sci. Technol. Hum. Values 35, 244–270. (10.1177/0162243909337121)

[30] Seafarers SD, Lavender S, Beaugrand G, Outram N, Barlow N, Crotty D, Evans J, Kirby R 2017 Seafarer citizen scientist ocean transparency data as a resource for phyoplankton and climate research. PLoS ONE 12, e0186092 (10.1371/journal.pone.0186092) 29211734 PMC5718423

[31] Poisson AC, McCullough IM, Cheruvelil KS, Elliott KC, Latimore JA, Soranna PA 2019 Quantifying the contribution of citizen science to broad-scale ecological databases. Front. Ecol. Environ. 18, 19–26. (10.1002/fee.2128)

[32] Danielsen F et al. 2014 Linking public participation in scientific research to the indicators and needs of international environmental agreements. Conserv. Lett. 7, 12–24. (10.1111/conl.12024)

[33] McGreavy B et al. 2017 The power of place in citizen science. Marine Policy Review 26, 94–95.

[34] Merenlender AM, Crall AW, Drill S, Prysby M, Ballard HL 2016 Evaluating environmental education, citizen science, and stewardship through naturalist programs. Conserv. Biol. 30, 1255–1265. (10.1111/cobi.12737) 27109290

[35] Kelly R, Fleming A, Pecl GT, Richter A, Bonn A 2019 Social licence through citizen science: a tool for marine conservation. Ecol. Soc. 24, 16 (10.5751/ES-10704-240116)

[36] Thiel M, Penna-Díaz MA, Luna-Jorquera G, Salas S, Sellanes J, Sotz W 2014 Citizen scientists and marine research: volunteer participants, their contributions and their projection for the future. Oceanogr. Mar. Biol. Annu. Rev. 52, 257–314.

[37] Andrachuk M, Marschke M, Hings C, Armitage D 2019 Smartphone technologies supporting community-based environmental monitoring and implementation: a systematic scoping review. Biol. Conserv. 237, 430–442. (10.1016/j.biocon.2019.07.026)

[38] Pocock MJO et al. 2018 A vision for global biodiversity monitoring with citizen science. Adv. Ecol. Res. 59, 169–223. (10.1016/bs.aecr.2018.06.003)

[39] Robinson LD, Cawthray-Syms JL, West SE, Bonn A, Ansine J 2018 Ten principles of citizen science. In Innovation in open science, society and policy (eds Hecker S, Hacklay M, Bowser A, Makuch Z, Vogel J, Bonn A), pp. 27–40. London, UK: UCL Press.

[40] McRobert CJ, Hill JC, Smale T, Hay EM, van der Windt DA 2018 A multi-modal recruitment strategy using social media and internet-mediated methods to recruit a multidisciplinary, international sample of clinicians to an online research study. PLoS ONE 13, e0200184 (10.1371/journal.pone.0200184) 29979769 PMC6034855

[41] Nishimura R, Wagner J, Elliott M 2015 Alternative indicators for the risk of non-response bias: a simulation study. Int. Stat. Rev. 84, 43–62. (10.1111/insr.12100) 27212786 PMC4871316

[42] Edwards R, Holland J 2013 What is qualitative interviewing? London, UK: Bloomsbury Academic Bloomsbury Publishing Plc UK.

[43] Lynch S 2018 OpenLitterMap.com—Open data on plastic pollution with blockchain rewards (Littercoin). Open Geospat. Data Softw. Stand. 3, 1 (10.1186/s40965-018-0050-y)

[44] Stuart-Smith RD et al. 2017 Assessing national biodiversity trends for rocky and coral reefs through the integration of citizen science and scientific monitoring programs. BioScience 67, 134–146. (10.1093/biosci/biw180) 28596615 PMC5384302

[45] McCoy E, Burce R, David D, Aca EQ, Hardy J, Labaja J, Snow SJ, Ponzo A, Araujo G 2018 Long-term photo-identification reveals the population dynamics and strong site fidelity of adult whale sharks to the coastal waters of Donsol, Philippines. Front. Mar. Sci. 5, 271 (10.3389/fmars.2018.00271)

[46] Fairclough DV, Brown JI, Carlish BJ, Crisafulli BM, Keay IS 2014 Breathing life into fisheries stock assessments with citizen science. Sci. Rep. 4, 7249 (10.1038/srep07249) 25431103 PMC5384193

[47] Lauro FM et al. 2014 The common oceanographer: crowdsourcing the collection of oceanographic data. PLoS ONE 12, e1001947 (10.1371/journal.pbio.1001947) PMC4159111 25203659

[48] Kelling S et al. 2019 Using semi-structured surveys to improve citizen science data for monitoring biodiversity. BioScience 69, 170–179. (10.1093/biosci/biz010) 30905970 PMC6422830

[49] Brenton P, von Gavel S, Vogel E, Lecoq M-E 2018 Technology infrastructure for citizen science. In Citizen science—innovation in open science, society and policy (eds Hecker S, Haklay M, Bowser A, Makuch Z, Vogel J, Bonn A), pp. 63–80. London, UK: UCL Press.

[50] Phillips TB, Ferguson M, Minarchek M, Porticella N, Bonney R 2014 User's guide for evaluating learning outcomes in citizen science. Ithaca, NY: Cornell Lab of Ornithology.

[51] Kieslinger B, Schäfer T, Heigl F, Dörler D, Richter A, Bonn A 2018 The challenge of evaluation: an open framework for evaluating citizen science activities. OSF. (https://osf.io/p5f29/#!)

[52] Robinson LM, Gledhill DC, Moltschaniwskyj NA, Hobday AJ, Frusher S, Barrett NS, Stuart-Smith J, Pecl GT 2015 Rapid assessment of an ocean warming hotspot reveals ‘high’ confidence in potential species' range extensions. Global Environ. Change 31, 28–37. (10.1016/j.gloenvcha.2014.12.003)

[53] Stuart-Smith J, Pecl G, Pender A, Tracey S, Villanueva C, Smith-Vaniz WF 2016 Southernmost records of two *Seriola* species in an Australian ocean-warming hotspot. Mar. Biodivers. 48, 1579–1582. (10.1007/s12526-016-0580-4)

[54] Lenanton RCJ, Dowling CE, Smith KA, Fairclough DV, Jackson G 2017 Potential influence of a marine heatwave on range extensions of tropical fishes in the eastern Indian Ocean—invaluable contributions from amateur observers. Reg. Studies Mar. Sci. 13, 19–31. (10.1016/j.rsma.2017.03.005)

[55] Edgar GJ, Bates AE, Bird TJ, Jones AH, Kininmonth S, Stuart-Smith RD, Webb TJ 2016 New approaches to marine conservation through the scaling up of ecological data. Annu. Rev. Mar. Sci. 8, 435–461. (10.1146/annurev-marine-122414-033921) 26253270

[56] Haklay M 2018 Participatory citizen science. In Citizen science—innovation in open science, society and policy (eds Hecker S, Haklay M, Bowser A, Makuch Z, Vogel J), pp. 52–62. London, UK: UCL Press.

[57] Theobald EJ et al. 2015 Global change and local solutions: tapping the unrealized potential of citizen science for biodiversity research. Biol. Conserv. 181, 236–244. (10.1016/j.biocon.2014.10.021)

[58] Martin VY, Smith L, Bowling A, Christidis L, Lloyd DJ, Pecl GT 2016 Citizens as scientists: what influences public contributions to marine research? Sci. Commun. 38, 495–522. (10.1177/1075547016656191)

[59] Ballard HL, Phillips TB, Robinson L 2018 Conservation outcomes of citizen science. In Citizen science—innovation in open science, society and policy (eds Hecker S, Haklay M, Bowser A, Makuch Z, Vogel J, Bonn A), pp. 254–268. London, UK: UCL Press.

[60] Isaac NJ, Strien AJ, August TA, Zeeuw MP, Roy DB 2014 Statistics for citizen science: extracting signals of change from noisy ecological data. Methods Ecol. Evol. 5, 1052–1060. (10.1111/2041-210X.12254)

[61] Isaac NJ et al. 2019 Data integration for large-scale models of species distributions. Trends Ecol. Evol. 35, 56–67. (10.1016/j.tree.2019.08.006) 31676190

[62] Ardoin NM, Bowers AW, Gaillard E 2020 Environmental education outcomes for conservation: a systematic review. Biol. Conserv. 241, 108224 (10.1016/j.biocon.2019.108224)

[63] Haklay M. 2013 Citizen science and volunteered geographic information—overview and typology of participation. In Crowdsourcing geographic knowledge: volunteered geographic information (VGI) in theory and practice (eds Sui D, Elwood S, Goodchild M), pp. 105–122. Dordrecht, The Netherlands: Springer.

[64] Cigliano JA, Ballard HL 2017 The promise of and the need for citizen science for coastal and marine conservation. In Citizen science for coastal and marine conservation (eds Cigliano JA, Ballard Heidi), pp. 3–16. London, UK: Routledge.

[65] Jordan R, Ballard HL, Phillips TB 2012 Key issues and new approaches for evaluating citizen-science learning outcomes. Front. Ecol. Environ. 10, 307–309. (10.1890/110280)

[66] Martin VY, Christidis L, Pecl GT 2016 Public interest in marine citizen science: is there potential for growth? BioScience 66, 683–692. (10.1093/biosci/biw070)

[67] Storksdieck M et al. 2016 Associations with citizen science: regional knowledge, global collaboration. Citizen Sci. Theory Pract. 1, 1–10. (10.5334/cstp.55)

[68] Bela G et al. 2016 Learning and the transformative potential of citizen science. Conserv. Biol. 30, 990–999. (10.1111/cobi.12762) 27185104

[69] Bonney R, Cooper CB, Dickinson J, Kelling S, Phillips TB, Rosenberg KV, Shirk J 2009 Citizen science: a developing tool for expanding science knowledge and scientific literacy. BioScience 59, 977–984. (10.1525/bio.2009.59.11.9)

[70] Hind-Ozan EJ, Pecl GT, Ward-Paige CA 2017 Communication and trust-building with the broader public through marine and coastal citizen science. In Citizen science for coastal and marine conservation (eds JA Cigliano, HL Ballard), pp. 261–278. London, UK: Routledge.

[71] Haywood BK 2015 Beyond data points and research contributions: the personal meaning and value associated with public participation in scientific research. Int. J. Sci. Educ. Part B. 6, 239–262. (10.1080/21548455.2015.1043659)

[72] Freitag A 2017 Factors determining participant quality in collaborative water quality research. Environ. Sociol. 3, 248–259. (10.1080/23251042.2017.1289592)

[73] Aceves-Bueno E et al. 2015 Citizen science as an approach for overcoming insufficient monitoring and inadequate stakeholder buy-in in adaptive management: criteria and evidence. Ecosystems 18, 493–506. (10.1007/s10021-015-9842-4)

[74] Gottschalk-Druschke C, Seltzer CE 2012 Failures of engagement: lessons learned from a citizen science pilot study. Appl. Environ. Educ. Commun. 11, 178–188. (10.1080/1533015X.2012.777224)

[75] Bennett NJ, DiFrance A, Calò A, Nethery E, Niccolini F, Milazzo M, Guidetti P 2019 Local support for conservation is associated with perceptions of good governance, social impacts, and ecological effectiveness. Conserv. Lett. 12, e12640 (10.1111/conl.12640)

