# Supplementary material for: Citizen science and marine conservation: a global review
Source: Philos Trans R Soc Lond B Biol Sci. 2020 Nov 2;375(1814):20190461. doi: 10.1098/rstb.2019.0461 (PMC7662190; doi:10.1098/rstb.2019.0461)
Supplement: Appendix A - Survey questionnaire. All questions of the online survey. [file rstb20190461supp1.docx]

**Appendix A**

Survey questions (launched on Survey Monkey July-August 2019)

**Intro**

Welcome to our Marine Citizen Science survey. We are excited to hear about your project, and to be able to include it in this research. We are a team of marine and citizen science researchers who aim to document and share useful results that can be used to inform and improve marine citizen science programmes around the world. Our objective is to document and understand global marine citizen science (topics, extent and potential) and to present an agenda for developing citizen science for conservation moving forward. In particular, we aim to achieve a realistic assessment of whether or not projects follow the **10 Principles of Citizen Science** (www.ecsa.citizen-science.net/engage-us/10-principles-citizen-science) in practice; whether the principles are useful, and/or what support might be required in developing them for the needs of marine citizen science programmes.

Importantly, all the project results will be **anonymous** so please **do share all details you think are relevant - both ‘positive’ as well as ‘negative’ experiences/results.** There will also be space for comments and additions where you want to share and suggest more ☺ If you have any further questions about this project please contact us via email at [r.kelly@utas.edu.au](mailto:r.kelly@utas.edu.au). Thank you!

Project info

1. Name of project
2. Website
3. Location
4. Partners, i.e. groups your project works with *(tick all that apply)*
   1. Government
   2. Environment agencies
   3. Universities
   4. Schools
   5. NGOS
   6. Marine research institutes
   7. Private enterprise
   8. Other (please identify)
5. Funders, i.e. bodies that your project is fully or partly supported by (*tick all that apply)*
   1. Government
   2. Environment agencies
   3. Universities
   4. Schools
   5. NGOS
   6. Marine research institutes
   7. Private enterprise
   8. Other (please identify)
6. Project focus. Tick all that apply.
   1. Ecosystem type *(tick all that apply – beaches and coasts, rocky seashore, coral reef, open ocean, mangroves, seagrass, other)*
   2. Method or approaches (*tick all that apply)*
      1. *Online (tick all that apply - website, mobile app, other tech)*
      2. *In person (tick all that apply – with individuals, with groups, project meet-ups, frequent project events, annual events other (please identify))*
   3. Data collected (*tick all that apply - on a specific location or environment, species identification, population counts, litter counts, other (please identify)*
   4. Education via (*tick all that apply - universities and/or schools, museums, website and/or online content, newsletters, in-person seminars, excursions, other (please identify)).*
7. Approx. number of participants
   1. Approx. number of actively participating participants
   2. Approx. number of participants signed up to your project (incl. active participants)
8. Do participants engage (tick all that apply - online, in person).
   1. How frequently does your project engage with participants? *(tick all that apply -sporadic events, weekly, monthly, annually, box for comment)*
9. 10 principles of citizen science

These 10 principles were identified by the European Citizen Science Association in 2015 to improve the practice of citizen science. However, currently there is no evidence as to whether these actually work to improve citizen science. In this section, we ask you to please honestly evaluate your response to these questions so that we can achieve a realistic assessment of whether projects follow the 10 principles in practice. Please remember that all responses will be anonymous. For more information on the 10 principles, see:

<https://ecsa.citizen-science.net/engage-us/10-principles-citizen-science>

1. What new knowledge and/or understanding has your project generated? *Why/how was this new? (Text box response)*
2. Does your project have a genuine science outcome? *If yes, please identify:*
   - 1. Data
     2. Museum collection
     3. Scientific journal publication
     4. Government or other reports
     5. Other (identify)
     6. We would be very grateful if you could please list the main outcomes identified above, or provide weblinks: (text box)
3. What benefit does your project provide for:
   - 1. the professional scientists *(tick all that apply - data collection, engagement with public, research question identification, knowledge exchange/learning, building trust, additional publications, other)*
     2. the citizen scientists *(tick all that apply - data collection, engagement with science, knowledge exchange/learning, address community concerns/issues, social network, publications, other).*
4. Were participants involved in the scientific process? *If yes, which stages of the scientific process were/are the citizen scientists involved?*

*(1-5; 1 = not involved – 5 = highly involved)*

- - 1. Identifying project objectives and agenda (with comment box)
    2. defining the research questions
    3. study or research design
    4. data collection
    5. data analysis
    6. reviewing/interpretation
    7. publication and/or reporting
    8. other

1. Are project feedbacks provided to the citizen scientists? *(tick all that apply - newsletter, website, social media, personal feedback, meetings, other).*
2. How does your project control for scientific quality? i.e. for the limitations and biases inherent in any form of scientific research. And what are your views on this? (text box)
   - 1. Beforehand (*tick all that apply - rigorous scientific design, joint design with community manager, in-person training, online training, other)*
     2. During (*tick all that apply -* trainer present, online *identification tools, printed training tools, participant surveys, standardised protocols, checklists, recording of survey metadata i.e. time, length of observation, location, etc., other)*
     3. After *(tick all that apply - data quality assurance with cross checks, photo ID, statistical modelling to account for uncertainties, other)*
3. Is the project data provided to
   1. the participants
   2. the public
   3. other scientists
   4. If yes, how?
4. Are the project results provided to
   1. the participants
   2. the public
   3. other scientists
   4. If yes, how?
5. Are project results published in open access formats? *If yes, where?*
6. Are the citizen scientists acknowledged in project outputs? (*Tick all that apply -* government reports, publications, websites, media articles, public events, other)
7. Are the projects outcomes evaluated?
   - 1. (*Tick all that apply - scientific outputs, data quality, participant experience, wider social/policy impact, other)*
     2. *How? Text box response.*
8. Are legal and ethical citizen science issues considered?
   - 1. *(Tick all that apply - copyright, IP, data-sharing, confidentiality, environmental impacts, other)*
     2. *How? (Can you please provide tips or templates?)*

Marine conservation goals and objectives

1. Is your project:
   1. Part of a larger conservation or management initiative? *If yes, please identify.*
   2. Part of a long-term monitoring programme? *If yes, please identify.*
   3. Collaborating with other organisations or initiatives to achieve shared conservation goals? *If yes, please identify.*
   4. Working with decision-makers to provide science for policy?
2. Does your project contribute or build upon current knowledge on marine biodiversity (i.e. species and/or environments)?

*If yes, how? (text box response)*

1. Does your project contribute to informed ocean management (i.e. of marine species and/or environments)?
   1. *If yes, how? And at what scales? (text box response)*
   2. *If no, is their potential for your project to contribute in the future? At what scales?*
2. Have the results/outcomes of your project been used to their maximum potential? *(text box response)*
   1. *Have you experienced any barriers to developing/implementing your project?*
   2. *If yes, please identify.*
3. Does your project promote participants’ interest and connection to the marine environment?

*If yes, how? (text box response).*

Thank you again for your very useful input and participation in this survey. If you wish to provide any additional information or comments, please do so here: _________________________________________

We intend to share all of the results of this research as they become available. **If you wish to receive this information, please provide your email here:** ________________________________________________

Finally - all results will protect the anonymity of your project. However, we do want to acknowledge and recognise this generous contribution from the marine citizen science community. We will include a list of all projects as part of our academic and online outputs. If you **do not wish** to be included in this list, please tick the box below.

If you have any further questions about this project please contact us via email at [r.kelly@utas.edu.au](mailto:r.kelly@utas.edu.au)

Thank you, and speak soon!
