# Supplementary material for: Citizen science and marine conservation: a global review
Source: Philos Trans R Soc Lond B Biol Sci. 2020 Nov 2;375(1814):20190461. doi: 10.1098/rstb.2019.0461 (PMC7662190; doi:10.1098/rstb.2019.0461)
Supplement: Appendix B - Summary of survey results. Additional figures and tables for analysis results for the Ten Principles. [file rstb20190461supp2.pdf]

Appendix B – Summary of survey results

Note: some qualitative responses have been omitted/edited to protect the anonymity of the survey participants.

i) Ten Principles of Citizen Science

Principle #1 - Citizen science projects actively involve citizens in scientific endeavour that generates new knowledge or understanding

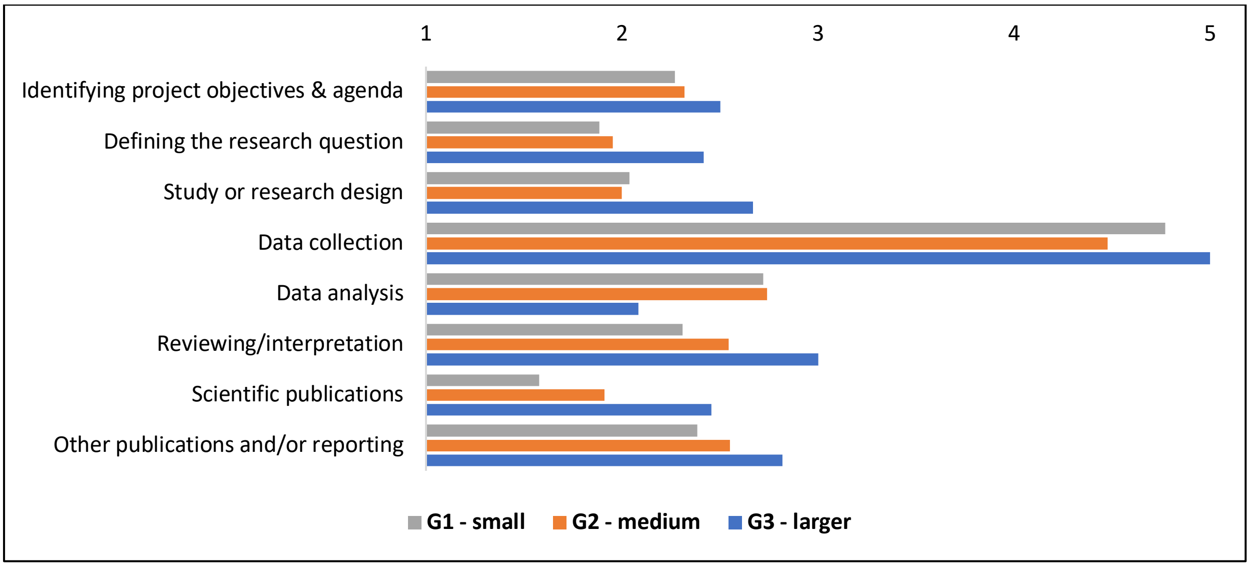

Figure B1. (Note, Figure 3 in manuscript). How were/are the citizen scientists involved in the scientific process? (1 = not involved; 5 = completely involved). These responses to the Likert data questions were averaged to give a continuous (i.e. interval) score for criteria (n=62; *G1=27, G2=22, G3=13*).

Principle #2 - Citizen science projects have a genuine science outcome

	G1 – small	G2- medium	G3 - larger
Yes	69%	96%	73%
No	3%	0%	7%

Figure B2. *Does your project have a genuine scientific outcome?* Percentage responses, according to project group sizes. The green colour indicates higher levels of feedback to citizen scientists, whilst the red indicates less feedback to citizen scientists.

Table B1. *Does your project have a genuine scientific outcome?* Qualitative responses.

It's early days yet, but the citizen science project is linked to major research projects in my lab on the topic of global change and biological responses in the ocean.
Citizen science data is included in scientific publications
We help communities describe the patterns they are interested in studying.
Our participants are collecting data to answer seven research questions. Each research question will become one scientific paper (e.g. Can immersive experiences change attitudes towards wetlands? What is the effect of nutrient enrichment on Blue Carbon stocks and fluxes? Etc.)
Mainly educational/nature observation
Much of the work will focus on increasing species distribution records but we will also be looking at intertidal species as water quality & climate indicators.

Test of a new methodology for monitoring marine benthic communities
Additional excursions are usually required in addition to the citizen science excursions to gather some types of data.
We are testing whether small scale restoration projects can assist the recovery of reefs at local scales
We're very short on species observations in South Africa, so data generated in these sorts of initiatives is always helpful
Data have been used in publications by others.
Depends on how you measure it
Species and habitat data (no further analysis carried out by citizen science, but the data is shared widely for further analysis by others)
Not in a science paper yet, but results reported through the media
We are able to use this data to see trends in where mussels are being found and use this info to plan our own investigations

Type of outcome	G1 – small	G2- medium	G3 - larger
Data	77%	92%	87%
Museum collection	3%	0%	7%
Scientific journal publication	40%	83%	47%
Government and other reports	37%	58%	40%
Data paper	17%	33%	20%

Figure B3. Percentage responses, according to project group sizes. The green colour indicates higher levels of feedback to citizen scientists, whilst the red indicates less feedback to citizen scientists.

Principle #3 - Citizen science provides benefits to both science and society.

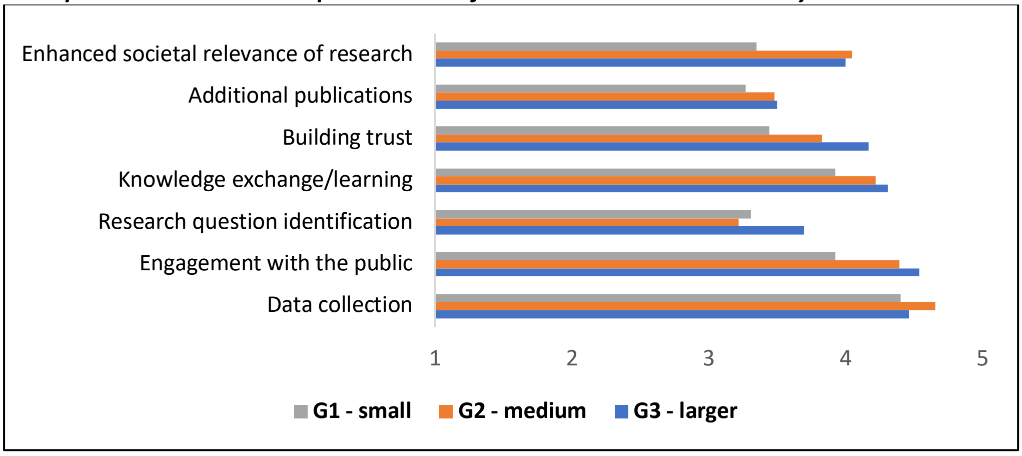

Figure B4. Scores for citizen science benefits for science (1 = no benefit; 5 = significant benefit). These responses to the Likert data questions were averaged to give a continuous (i.e. interval) score for criteria.

Principle #4 - Citizen scientists may participate in various stages of the scientific process.
This principle is related to i) *how were/are the citizen scientists involved in the scientific process?* (responses documented under #1 above - Figure B1) and also ii) *what methods/approaches are used to engage citizen scientists?* (below – Figure B5).

**Methods/approaches**	**G1 – small**	**G2- medium**	**G3 - larger**
Online - website	63%	83%	93%
Online - mobile app	20%	17%	67%
Online - social media	57%	75%	67%
In person - with individuals	49%	54%	67%
In person - with groups	63%	63%	80%
In person - project meet-ups	14%	33%	33%
In person - frequent project events	17%	21%	20%
In person - annual events	17%	21%	40%

Figure B5. Percentage responses, according to project group sizes. The green colour indicates higher levels of feedback to citizen scientists, whilst the red indicates less feedback to citizen scientists.

Principle #5 - Citizen scientists receive feedback from the project.

	**G1 – small**	**G2- medium**	**G3 - larger**
Newsletter	23%	75%	60%
Website	43%	83%	80%
Social media	54%	83%	67%
Personal feedback	31%	33%	27%
Meetings	20%	46%	27%

Figure B6. Percentage responses, according to project group sizes. The green colour indicates higher levels of feedback to citizen scientists, whilst the red indicates less feedback to citizen scientists.

Principle #6 - Citizen science, as with all forms of scientific inquiry, has limitations and biases that should be considered and controlled for.

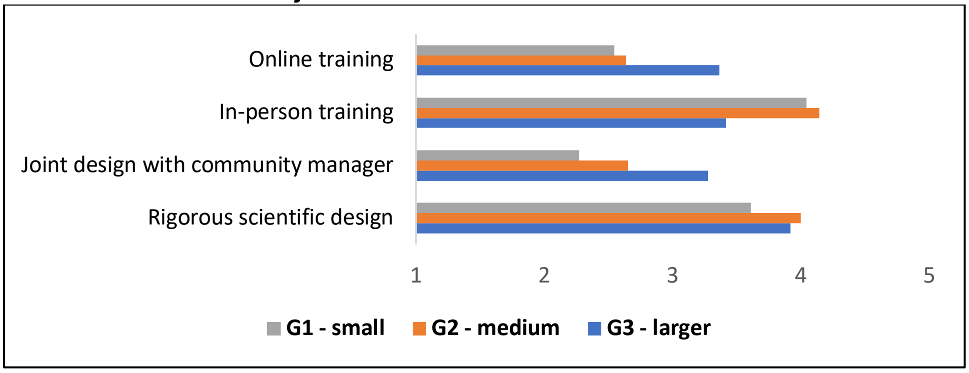

Figure B7. Scores of projects' control for scientific quality *before* the scientific activity (1 = no control; 5 = strong control). These responses to the Likert data questions were averaged to give a continuous (i.e. interval) score for criteria.

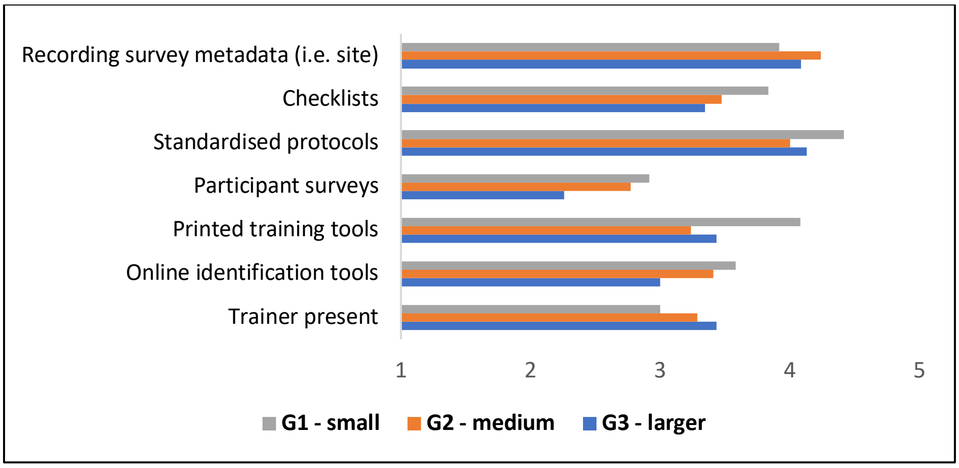

Figure B8. Scores of projects' control for scientific quality *during* the scientific (1 = no control; 5 = strong control). These responses to the Likert data questions were averaged to give a continuous (i.e. interval) score for criteria.

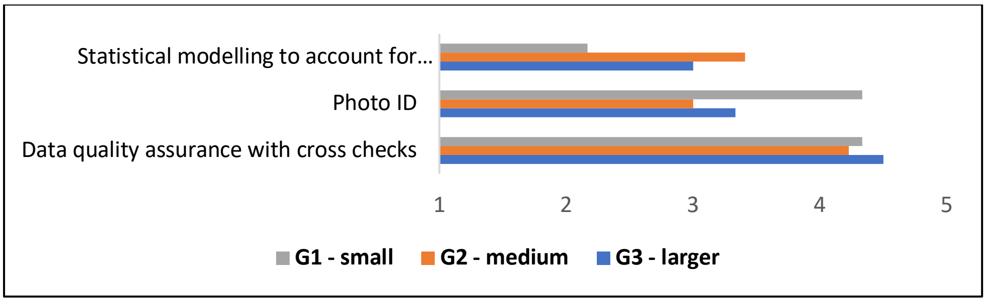

Figure B9. Scores of projects' control for scientific quality *after* the scientific (1 = no control; 5 = strong control). These responses to the Likert data questions were averaged to give a continuous (i.e. interval) score for criteria.

Principle #7 - Project data/meta-data are publicly available and results are published open-access

	G1 – small	G2- medium	G3 - larger
Yes	49%	63%	67%
No	20%	29%	7%

Figure B10. *Are project results published in open access formats?* Percentage responses, according to project group sizes. The green colour indicates higher levels of feedback to citizen scientists, whilst the red indicates less feedback to citizen scientists.

Table B2. *Who else is the project data provided to?* Qualitative responses.

Data is public and available in the web. Shared with the scientific community in meetings and publications
our data is will be available to all via the National Biodiversity Data Centre
It's freely available to anyone who wants it
Government
It is publicly available data and we do in-person presentations and newsletters with our volunteers to share results.
Government/enforcement agencies
The project data are not publicly available yet, but we intend to provide them for all the above and also for MPA managers and stakeholders.
Not the raw data, only summarised analysed versions
Open data
It's available for anyone on iNaturalist as far as I know, no limitations other than in the case of sensitive species
100% Open
The data is open and available to all

Management entities (State, federal, and tribal)
Publicly available on the website.
Press releases
Government agencies
Interest groups e.g. local group, local port authority
Fisheries Department
Academy of Science
Government
Anyone.
Government bodies, other NGOs and research institutions
It is publicly available to anyone that wishes to use it.

Principle #8 - Citizen scientists are suitably acknowledged by projects.

	G1 - small	G2 - medium	G3 – large
Government reports	9%	46%	40%
Scientific journal publications	20%	75%	60%
Website/online content	40%	63%	73%
Media articles	29%	54%	60%
Public events	26%	54%	33%

Figure B11. *How/where are citizen scientist contributions acknowledged?* Percentage responses, according to project group sizes. The green colour indicates higher levels of feedback to citizen scientists, whilst the red indicates less feedback to citizen scientists.

Table B3. *How/where are citizen scientist contributions acknowledged?* Qualitative responses.

Many people are anonymous, we acknowledge who we can.
At present each recorder is acknowledged via our mapping system and each recorder can access and explore their own data via our citizen science portal.
They will be in the acknowledgements section of all journal articles
There are too many volunteers to list, they are acknowledged openly but also anonymously.
Social media
They were acknowledged on the dissertation and will be in all the next publications we may have.
All acknowledged by community group
Name of surveyor is logged with the survey
Annual reports
Not individually. Their name is on the record, but there is a CC license associated with data submission.
As authors of the field guides
Participants are acknowledged as citizen scientists generally. Only on occasion are individuals acknowledged by name.
Exhibits
Project report
Each contributor is identified by their iNaturalist handle, which may or may not use their name. But, all iNat data is public and open source
Citizen scientists will be acknowledged in any scientific publication where their contribution was utilized.

Principle #9 - Citizen science programmes offer a range of benefits and outcomes which should be acknowledged and considered in project evaluation.

	G1 - small	G2 - medium	G3 – large
yes	51%	75%	60%
no	17%	8%	7%

Figure B12. *Are project outcomes evaluated?* Percentage responses, according to project group sizes. The green colour indicates higher levels of feedback to citizen scientists, whilst the red indicates less feedback to citizen scientists.

	G1 - small	G2 - medium	G3 – large
Scientific outputs	37%	67%	40%
Data quality	40%	63%	53%
Participant experience	29%	58%	27%
Wider social/policy impact	17%	54%	33%

Figure B13. *How are the project outcomes are evaluated?* Percentage responses, according to project group sizes. The green colour indicates higher levels of feedback to citizen scientists, whilst the red indicates less feedback to citizen scientists.

Principle #10 - Leaders of projects address legal & ethical considerations of the project.

	G1 - small	G2 - medium	G2 - larger
Copyright	20%	21%	40%
IP	11%	29%	40%
Data-sharing	40%	58%	60%
Confidentiality	26%	71%	60%
Environmental impacts	37%	50%	53%

Figure B14. *What kinds of legal and ethical issues does your project consider?* Percentage responses, according to project group sizes. The green colour indicates higher levels of feedback to citizen scientists, whilst the red indicates less feedback to citizen scientists.

Table B4. *What other kinds of legal and ethical issues are considered?* Qualitative responses.

Protection of citizen's private information; we needed ethics clearance
These are all considered, and it is a challenge to balance the community needs and individual needs. For example, if individuals wanted to share their data publicly but they don't live were their data are collected (e.g., tourists) then they don't know the vulnerabilities of the community. These decisions need to be made locally.
special tool for anonymous field data reporting
GDPR applies!
Animal ethics
Safety and Insurance
Safety
All processes involving citizens in the pilot project were submitted and approved by the ethics committee of the University. All citizens were informed and they authorized the use of the data they produced. They can also withdraw their authorization anytime.
Dive safety
Animal ethics
Animal safety and security
We make sure that divers understand they must be certified (and comfortable) taking photographs at that depth.
Diver safety is emphasized
Indigenous Consultation

ii) Contribution to marine management

	G1 - small	G2 - medium	G2 - larger
Yes	63%	71%	73%
No	3%	8%	7%

Figure B15. *Does your project contribute to informing ocean management? (i.e. of marine species and/or environments)?* Percentage responses, according to project group sizes. The green colour indicates higher levels of feedback to citizen scientists, whilst the red indicates less feedback to citizen scientists.

Table B4. *How does your project contribute to informing ocean management?* Qualitative responses.

Provide information to relevant stakeholders whenever possible.
CITES, Redlist, Fisheries management, Shark Sanctuaries, MPA monitoring
The program aims to better understand the importance of coastal Blue Carbon systems to leverage on their protection and restoration.
In the long-run, data will be publicly available and inform about plankton distribution in the global ocean.
Better understanding of microbial diversity and anthropogenic impact
Use of MPA's, population level monitoring, habitat shift and usage.
Marine litter data are collected, research drifters are recorded for the University
Increasing availability of species data and establishing a baseline data set.
New data for poorly documented species
Perhaps - depends on what happens with our reports when they get sent
Coastal environments and sediment transport
By defining the plastic pollution current status and baseline levels
evaluating the species-specific efficacy of reserves for increasing density and size
Data are being used in the 2022 MPA management review
Aiming to provide reliable data to management of the MPAs
Management of exploited fish populations in Western Australia
Restoration of coral reefs - management is very involved from scientific and policy perspectives on determining the future of the project and project goals
Collaboration with management agencies to discuss policies and procedures for active reef restoration. Review draft policies on the areas of interest to management.
Open data
Data included on national biodiversity databases
Data is used to inform policy and species/environment management
Provide input to local governing bodies
Provide big-picture view of jellyfish population dynamics, which currently cannot be quantified.
Records of species observations
MPA designation (e.g. ban on bottom fishing gear on seagrass beds)
By mapping areas with noise pollution problems
Basic knowledge of invasion status (presence and spread) is key to effective management for this invasive species. Data collected by CitSci volunteers are used to guide management actions, including removal efforts and regulations to prevent further spread.
Data is accessed by scientists and government agencies and used in reporting.
Gathering data of benthic marine species presence and cover percentage through time
Shells as symbol for ocean biodiversity management
For example, and octopus was one of the new species first detected, and now it is reported by fishers separately
Monitoring of specific sites
IUCN Red List Assessment
By carrying out the programme, participants feel very much connected to a proper ocean management
Providing scientific surveys of local artificial reefs
We hope that ocean managers will use this data to formulate conservation policies which will benefit shark population management
Data used at local, state, federal level for policy
The data is open
Changes in policy and law
Through data on global marine primary productivity
It informs marine managers of changes occurring in reef ecosystems over the long-term
